# Supplementary material for: The Effects of Artificial Diets on the Expression of Molecular Marker Genes Related to Honey Bee Health
Source: Int J Mol Sci. 2024 Apr 12;25(8):4271. doi: 10.3390/ijms25084271 (PMC11049949; doi:10.3390/ijms25084271)
Supplement: Supplementary file 1 [file ijms-25-04271-s001.zip › ijms-2945124-SI.pdf]

Supplementary material

The Effects of Artificial Diets on the Expression of Molecular Marker Genes Related to Honey Bee Health

Olga Frunze [1,2], Hyunjee Kim [1,2], Jeong-Hyeon Lee [1,2], and Hyung-Wook Kwon [1,2,*]

[1] Department of Life Sciences, Incheon National University, 119 Academy-ro, Yeonsu-gu, Incheon 22012, Republic of Korea; frunzeon@gmail.com (O.F.); jeonghyeon@inu.ac.kr (J.-H.L.)

[2] Convergence Research Center for Insect Vectors (CRCIV), Incheon National University, 119 Academy-ro, Yeonsu-gu, Incheon 22012, Republic of Korea

* Correspondence: hwkwon@inu.ac.kr; Tel.: +82-10-3379-6727

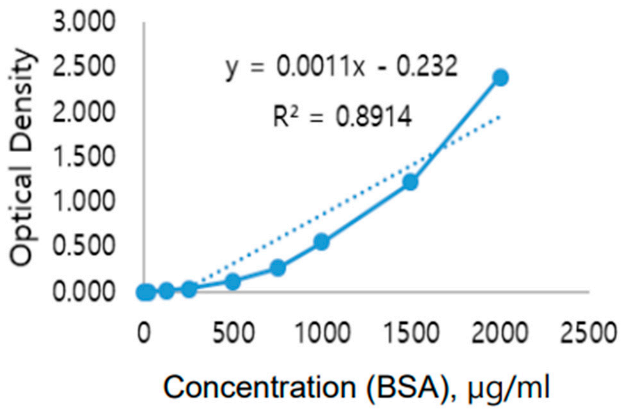

Figure S1. Calibration curve for the colorimetric method using the Pierce™ BCA Protein Assay Kit. BSA stands for Bovine Serum Albumin, and BCA stands for BiCinchoninic Acid.

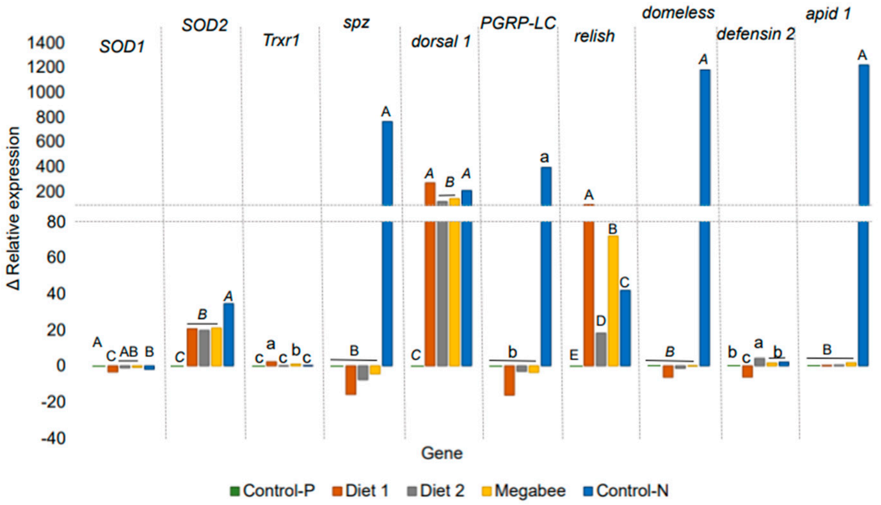

Figure S2. Differences in relative gene expression of defense system genes in honey bees under dietary conditions. Difference values were calculated by subtracting the relative gene expression in honey bees after dieting from that before dieting. One-way ANOVA and Duncan post hoc tests ($p < 0.05$) were conducted and marked in the chart with upper or lowercase letters.

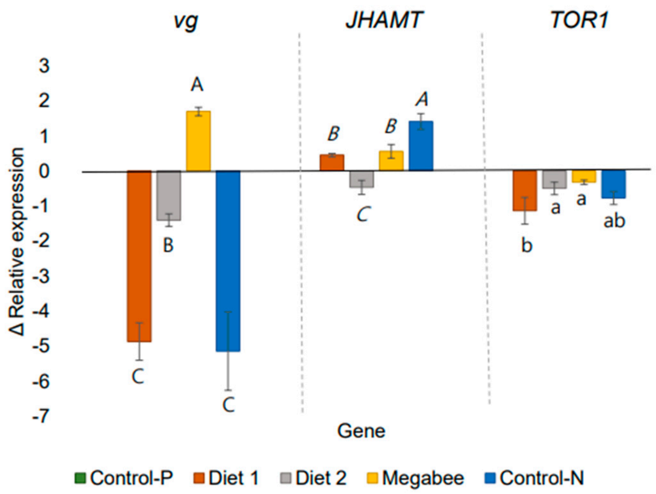

Figure S3. Differences in relative gene expression of nutrition markers in honey bees under dietary conditions. Difference values were calculated by subtracting the relative gene expression in honey bees after dieting from that before dieting. One-way ANOVA and Duncan post hoc tests ($p < 0.05$) were conducted and marked in the chart with upper or lowercase letters.

Table S1 PCR primer sequences.

	Locus	5`-3`	Primers	Amplicon length, bp	NCBI Reference Sequence	Efficiency (%)
1	*defensin-2*	F	ACCGCTGCTACCACTACGACA	139	NM_001011638.1	110.
2		R	GCCATTTCTGCAACTACCGCCT			
3	*relish*	F	TCCATTGCATGCAGCACTTCG	264	XM_026444175.1	97
4		R	ACACATGCACCAGCTTCAGGA			
5	*dorsal-1*	F	TGCAGCAAGTGGAACAACCAGT	114	XM_006566999.3	92
6		R	CAGGCCTACCTGCACCGAGA			
7	*domeless*	F	GCCGCTGCTCTTTGGCATCT	238	XM_006567690.3	108
8		R	GCCAAATTGTTGTTCCAACAGCCC			
9	*apid-1*	F	TTGTTGTTACCTTTGTAGTCGCGGT	70	NM_001011642.1	110
10		R	AGGCGCGTAGGTCGAGTAGG			
11	*PGRP-LC*	F	TGCAATGCGATGGCGACACA	105	XM_026441962.1	110
12		R	AGCGACTTGAGCACACCACAC			
13	*spz (spaetzel)*	F	TGGACGACAGCCCTCTTTGTCA	371	XM_006565534.3	92
14		R	GCGCCTTCGACGTGACGATT			
15	*SBV*	F	GTGGAACCCGAGTGTTTTGTAACCC	156	KY273489.1	98
16		R	AAGCTAAAAGCGTCCACTCTGTACTCT			
17	*DWV*	F	TGT GAA GTG GCG GAC GTT ACA GA	211	KT215904.1	100
18		R	GTA TTC TGG ACC CCA TCC GAA TGC			
19	*β-actin*	F	GGATTCCTATGTTGGTGATGAAGCCC	177	NM_001185145.1	100
20		R	GGTGCCTCAGTAAGAAGTACCGGATG			
21	*SOD1*	F	GCAGTGTGCGTTCTTCAGGGT	86	NM_001178027.1	99
22		R	TGACCGGTGACCTTCACGGA			
23	*SOD2*	F	GGCGGTAAACCAGACGCTGC	126	NM_001178048.2	110
24		R	TCCAAGCCAACCCCAACCAGA			
25	*Trxr-1*	F	CCTGTTGCTATACATGCGGGTCG	141	XM_006563201.3	110
26		R	TGCTGCTTCTTCGCTAAGGCCA			
27	*vg (vitellogenin)*	F	GCA GAA TAC ATG GAC GGT GT	146	NM_001011578.1	
28		R	GAA CAG TCT TCG GAA GCT TG			92.36
29	*JHAMT*	F	TTG GAC ATA GGT TGC GGA CC	302	NM_001327967.1	
30		R	AAT CCT TTT CCT CCT GGC CG			89.20
31	*TOR1*	F	AAC AAC TGT TGC TGA CGG TG	153	XM_006566642.3	
32		R	GTT GCA GTC CAG GCT TTT TG			90.54
